# Successful Thromboembolectomy for PTE in an Adolescent Patient With Catastrophic APS and SLE

**DOI:** 10.1002/ccr3.73911

**Published:** 2026-11-09

**Authors:** Azadeh Zeinab Mirzaee, Bita Paknejad, Aziz Eghbali, Reza Shiari, Niku Rahimi Afzal

**Affiliations:** ^1^ Pediatric Respiratory Diseases Research Center (PRDRC), National Research Institute of Tuberculosis and Lung Diseases (NRITLD), Masih Daneshvari Hospital, Faculty of Medicine Shahid Beheshti University of Medical Sciences Tehran Iran; ^2^ Pediatric Respiratory Diseases Research Center (PRDRC), National Research Institute of Tuberculosis and Lung Diseases (NRITLD), Masih Daneshvari Hospital, Faculty of Medicine, Masih Daneshvari Hospital Shahid Beheshti University of Medical Sciences Tehran Iran; ^3^ Clinical Research Development Center of Aliasghar Hospital Iran University of Medical Sciences Tehran Iran; ^4^ Department of Pediatric Rheumatology, Mofid Children's Hospital Shahid Beheshti University of Medical Sciences Tehran Iran

**Keywords:** antiphospholipid syndrome, catastrophic antiphospholipid syndrome, lupus anticoagulant, rivaroxaban, systemic lupus erythematosus, warfarin

## Abstract

In a 14‐year‐old with antiphospholipid syndrome (APS) and lupus anticoagulant, improper switching from warfarin and low‐dose aspirin to rivaroxaban led to life‐threatening recurrent thrombosis, pulmonary embolism, and pulmonary hypertension. Prompt return to standard anticoagulation plus immunosuppression stabilized the condition. This case underscores that DOACs are contraindicated in high‐risk APS.

## Key Clinical Message

In adolescent SLE and triple‐positive APS, switching from warfarin to rivaroxaban can trigger life‐threatening recurrent thrombosis and pulmonary embolism. DOACs are contraindicated in high‐risk APS. Timely resumption of warfarin, immunosuppression, and thromboembolectomy can be lifesaving.

## 1 Introduction

Venous thromboembolism (VTE) is a relatively uncommon occurrence in the pediatric population; however, when it does occur, it frequently signifies an underlying pathogenic process or systemic disorder that necessitates meticulous evaluation [1, 2]. Venous thromboembolism (VTE) is a relatively uncommon occurrence in the pediatric population; however, when it does occur, it frequently signifies an underlying pathogenic process or systemic disorder that necessitates meticulous evaluation [3]. Approximately 10%–20% of SLE patients are affected during childhood, often manifesting more severe clinical symptoms and adverse outcomes compared to adults [4, 5]. Antiphospholipid syndrome (APS) is a prothrombotic autoimmune disorder that can occur in conjunction with systemic lupus erythematosus (SLE) [6]. Antiphospholipid antibodies (aPLs)—encompassing anticardiolipin, anti‐β2 glycoprotein I, and lupus anticoagulant (LAC)—have been demonstrated to activate the endothelium and coagulation system, thereby increasing the risk of thrombosis. In the pediatric population, the most prevalent manifestation of antiphospholipid syndrome (APS) is venous thrombosis [7, 8].

Among aPLs, lupus anticoagulant (LAC) has been identified as the strongest predictor of thrombotic events, both in adults and children. The number of reports related to the severe manifestation of APS in children is limited, and the management of these patients is challenging, especially when the disorder leads to recurrent thrombosis [9].

Recurrent thrombosis constitutes a significant problem in patients with antiphospholipid syndrome (APS), particularly those with lupus anticoagulant (LAC) positivity. The administration of appropriate anticoagulation management is imperative for these patients. Standard treatments include the use of warfarin or heparin with target INR guidance. However, there is limited data suggesting that caution has been raised against the use of direct oral anticoagulants (DOACs), such as rivaroxaban, in patients with antiphospholipid syndrome (APS), particularly in high‐risk subgroups [10, 11].

In patients with more severe and life‐threatening manifestations, including cases approaching CAPS (Catastrophic Antiphospholipid Syndrome), a multimodal therapeutic approach is recognized as an enhanced treatment strategy. This approach includes the use of anticoagulants, suppression of inflammation with corticosteroids, plasmapheresis and/or gammaglobulin, and immunomodulatory therapies [12].

The present study reports on a case of a 14‐year‐old female patient with systemic lupus erythematosus (SLE) and a history of recurrent venous thromboembolism (VTE) who presented with severe capillarity‐associated pleural effusion syndrome (CAPS)‐like clinical manifestations. The significance of this report lies in its provision of a clinical example of successful treatment, including the administration of anticoagulant interventions, immunomodulatory therapies, and appropriate aggressive measures, which resulted in the stabilization of the patient's clinical condition. This case exemplifies that in young patients with severe APS, comprehensive treatment and proper management of anticoagulant therapy can yield substantial improvement outcomes even in the context of augmented risks of recurrent thrombosis.

## 2 Case History/Examination

The patient was a 14‐year‐old female with a body mass index (BMI) in the obese range who had no known family history of thrombosis. Two months prior to the current presentation, she had been hospitalized for right lower extremity deep vein thrombosis (DVT) and acute pulmonary embolism (PTE), and was discharged on warfarin. During the same hospitalization, a positive lupus anticoagulant (LA) test was reported once, and the test was scheduled to be repeated after 13 weeks to confirm the diagnosis of antiphospholipid syndrome (APS).

Six days prior to the current presentation, despite the positive LA test result, the patient's treatment was inappropriately changed from warfarin and low‐dose aspirin to rivaroxaban without adequate specialist supervision. Following the alteration in medication, the patient exhibited a progressive exacerbation of dyspnea, weakness, dizziness, fever, and a worsening cough. This indicated an absence of efficacy in the anticoagulant therapy and the occurrence of recurrent thrombosis.

Upon admission, the patient exhibited tachycardia (pulse rate of 130 beats per minute), fever (temperature of 37.7°C), and severe respiratory distress. The patient's blood pressure was documented at 100/60 mmHg. The patient was promptly admitted to the pediatric intensive care unit (PICU).

## 3 Differential Diagnosis, Investigations and Treatment

### 3.1 Investigations

Laboratory findings indicated an active inflammatory and coagulation syndrome, with evidence of multisystem involvement. The patient exhibited inflammatory and hematological indices indicative of severe anemia (hemoglobin [Hb]: 7.7 g/dL) with thrombocytopenia (platelet [Plt]: 65,000), leukocytosis (17,800 white blood cells [WBC]), and marked elevation of inflammatory markers (C‐reactive protein [CRP]: 56, Erythrocyte Sedimentation Rate [ESR]: 80). Coagulation and thrombophilia assessment revealed elevated D‐dimer levels (4000) and an INR within the range of 2.1 in a patient who had been administered rivaroxaban for 6 days. The patient exhibited a triple‐positive profile (LA, Anti‐Cardiolipin > 100, and Anti‐beta2 GP1 > 100), which is widely regarded as a high‐risk profile for APS. The patient also exhibited signs of acute kidney injury (BUN 60 mg/dL, Cr 1.5 mg/dL), hypoalbuminemia (albumin 2.8 g/dL), and a reticulocyte count of 1.5% with a negative Direct Coombs test. ANA was positive, supporting the diagnosis of evolving SLE. Leg Doppler ultrasound showed acute DVT in the left femoral vein, suggesting recurrent VTE on a different side from the initial involvement. CT pulmonary angiography revealed thrombosis in the main pulmonary artery with an acute‐on‐chronic pattern of peripheral embolism (PTE) and pulmonary artery pressure > 40 mmHg, indicating chronic thromboembolic pulmonary hypertension (CTEPH).

Representative axial views of the CTPA demonstrate irregular filling defects within the pulmonary vasculature, confirming extensive acute‐on‐chronic thromboembolism (Figure 1).

**FIGURE 1:**
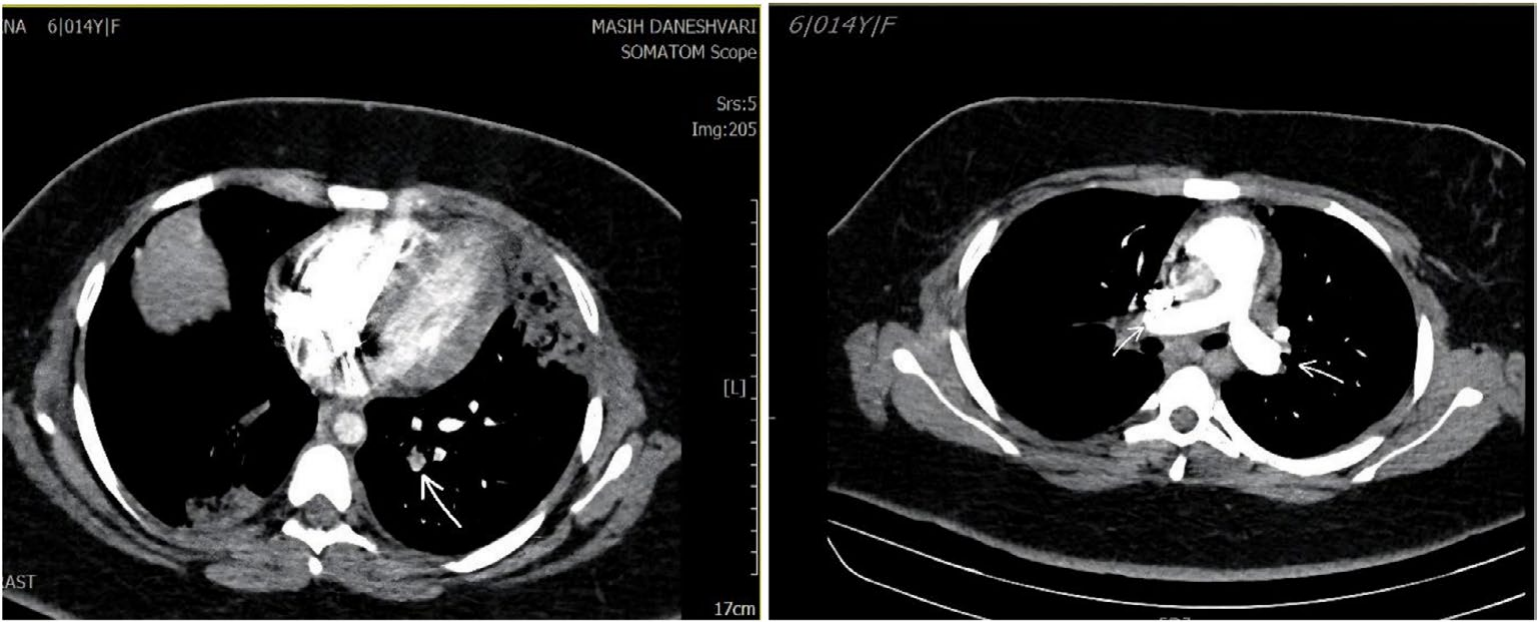
Axial views of computed tomography pulmonary angiography (CTPA). The images demonstrate irregular filling defects within the main and peripheral branches of the pulmonary arteries (white arrows), consistent with acute‐on‐chronic pulmonary thromboembolism.

A kidney biopsy demonstrated membranous glomerulonephritis, Stage I‐II, with substantial proteinuria (1026 mg/day) and a protein/creatinine ratio of 1.3, substantiating a diagnosis of lupus nephritis with APS nephropathy.

Table 1 presents a synopsis of the clinical and paraclinical observations concerning the patient.

**TABLE 1:** Summary of clinical and paraclinical findings.

Section	Key findings	Interpretation and significance
Demographics	14‐year‐old obese female	SLE and VTE in pediatric/adolescent populations; obesity is a known thrombotic risk factor.
Past history	DVT/PTE 2 months prior; single positive Lupus Anticoagulant (LA) test	History of thrombosis and preliminary evidence of antiphospholipid syndrome (APS).
Trigger Event	Inappropriate switch from warfarin to rivaroxaban 6 days prior	Anticoagulation failure in a high‐risk, LA‐positive patient.
Presenting Symptoms	Dyspnea, fever, malaise, and cough	Acute presentation of recurrent pulmonary thromboembolism (PTE) and inflammatory syndrome.
Examination (PICU)	Pulse 130, BP 100/60, Temp 37.7°C, respiratory distress	Signs of potential shock, tachycardia, and severe PTE.
CBC	Hb: 7.7 g/dL, Plt: 65 × 10^3^/μL, WBC: 17.8 × 10^3^/μL	Anemia, thrombocytopenia, leukocytosis (indicative of inflammation and active SLE).
Coagulation/Inflammation	CRP: 56 mg/L, ESR: 80 mm/h, D‐dimer: 4000 ng/mL	Markers of acute inflammatory syndrome and high thrombotic activity.
APS Serology	LA positive, Anti‐β_2_GP1 > 100 U, Anti‐Cardiolipin > 100 U	High‐risk triple‐positive APS profile.
SLE Markers	ANA positive, Cr: 1.5 mg/dL, Alb: 2.8 g/dL, Proteinuria: 1026 mg/day	Evolving SLE with renal involvement (lupus nephritis).
Imaging	Acute left femoral DVT (new), CTPA: acute‐on‐chronic PTE, PAP > 40 mmHg	Recurrent VTE, emerging chronic thromboembolic pulmonary hypertension (CTEPH), extensive pulmonary vascular involvement.
Renal Biopsy	Membranous glomerulonephritis, Stage I‐II	Histological confirmation of lupus nephritis and potential APS nephropathy.
Final Diagnosis	SLE, triple‐positive APS, catastrophic APS‐like presentation	Life‐threatening manifestation driven by active SLE/APS and suboptimal management.

### 3.2 Differential Diagnosis

The differential diagnosis included catastrophic antiphospholipid syndrome (CAPS), thrombotic thrombocytopenic purpura (TTP), disseminated intravascular coagulation (DIC), and sepsis‐induced coagulopathy. However, the triple‐positive APS profile, histopathological evidence of lupus nephritis, and absence of severe microangiopathic hemolytic anemia favored catastrophic APS‐like manifestation secondary to SLE.

### 3.3 Treatment

The treatment plan encompassed supportive care in the PICU, administration of antibiotics, cell therapy, and reinitiation of anticoagulation with intravenous heparin. The therapeutic approach was modified to warfarin with a target INR of 2–3. Additionally, pulse methylprednisolone therapy and immunosuppressive therapy were initiated in accordance with the renal biopsy findings.

## 4 Conclusion and Results (Outcome and Follow‐Up)

The patient was discharged with a stable clinical condition after receiving appropriate treatments. Follow‐up was planned for monitoring of anticoagulation, renal function, and disease activity of SLE and APS.

## 5 Discussion

The present case report presents a challenging clinical case of a 14‐year‐old girl with evolving systemic lupus erythematosus (SLE) and triple‐risk antiphospholipid syndrome (APS) who presented with severe recurrent venous thromboembolism (VTE) and catastrophic APS‐like presentation. The severe manifestation of the condition was attributed to a critical management error in the patient's anticoagulant therapy.

A pivotal element in the genesis of this complication was the erroneous transition from anticoagulant therapy to rivaroxaban (a direct factor Xa inhibitor). The utilization of direct oral anticoagulants (DOACs), such as rivaroxaban, in patients diagnosed with antiphospholipid syndrome, particularly those who are triple‐positive or have positive lupus anticoagulants, is not recommended based on established guidelines and research evidence. This recommendation is attributed to a substantially elevated risk of recurrent thromboembolism. Consequently, warfarin, with a target International Normalized Ratio (INR) of 2–3, is regarded as the prevailing therapeutic standard [13, 14, 15, 16].

The rarity of severe manifestations of APS in children, as well as the overlap with SLE, constitutes a major focus of this report. As also noted in related articles, SLE is an important factor in increasing the risk of thrombosis [17].

The patient's case exhibited not only systemic lupus erythematosus (SLE) but also triple‐positive antiphospholipid antibodies (APS), which are associated with the highest risk of recurrent thrombosis.

The present case is analogous to that documented by Liu et al. [17] wherein the patient initially presented with nephrosis and venous embolism, and was diagnosed with SLE/APS 5 years later. The two cases illustrate that APS in children frequently manifests earlier than the definitive diagnosis of SLE and can be linked to severe vascular and renal manifestations (such as proteinuria and membranous glomerulonephritis in the present cases). However, our case, in contrast to the case reported by Jimenez et al. [14] whose initial presentation was arterial thrombosis, exhibited the more classic presentation of venous thrombosis (VTE), albeit with a significantly higher severity level.

Furthermore, a recent report by Tekinhatun and Duyu [18] described a 17‐year‐old male with APS who presented with multisystemic thrombotic events, including cerebral venous sinus thrombosis, pulmonary embolism, and intracardiac thrombi. While both cases highlight the severity of pediatric APS and the critical role of multimodal imaging, they exhibit notable differences. The case reported by Tekinhatun and Duyu involved primarily arterial and unusual cardiac thromboses with a persistently positive LA but negative ANA and anticardiolipin antibodies. In contrast, our patient exhibited a classic venous thromboembolism (VTE) presentation with a highly aggressive triple‐positive antibody profile (LA, anti‐cardiolipin, and anti‐β2GP1), alongside evolving SLE and lupus nephritis. Moreover, while the previous case emphasizes the diagnostic value of cardiac MRI and echocardiography for intracardiac thrombi, our case underscores the catastrophic consequences of inappropriate anticoagulation (switching from warfarin to rivaroxaban) in a high‐risk triple‐positive APS patient. Together, these reports expand the recognized spectrum of severe pediatric APS, highlighting that both arterial and venous catastrophic presentations require early, aggressive, and guideline‐directed multidisciplinary management.

The significance of timely diagnosis of SLE in such cases cannot be overstated. The presence of significant proteinuria, cytopenia, and triple APS in our patient underscores the urgent need for immediate evaluation and diagnosis of SLE, as the manifestation of catastrophic APS is a fatal and often preventable complication.

The primary objective of this case study is to examine the efficacy of the treatment and its impact on patient survival. The patient was in a life‐threatening condition at the time of presentation. The patient exhibited severe dyspnea, respiratory distress, hypotension, and a history of recurrent thrombosis. These symptoms were suggestive of acute pulmonary embolism (PTE) on a chronic basis and imminent risk of organ failure. The patient's condition was stabilized by the following key measures: the rapid initiation of immunosuppressive therapy with pulse methylprednisolone to control inflammation and SLE activity, the reinstatement of anticoagulation to warfarin with a target International Normalized Ratio (INR) of 2–3, and careful management in the pediatric intensive care unit (PICU).

Furthermore, due to severe PTE, the patient underwent pulmonary endarterectomy, a critical intervention aimed at reducing pulmonary artery pressure, enhancing pulmonary blood flow, and averting CTEPH and organ failure. This surgical intervention, in conjunction with adequate anticoagulant therapy and immunosuppression, was instrumental in preserving the patient's life and averting fatal complications. From the standpoint of anticoagulant therapy, the inappropriate transition from warfarin to rivaroxaban a mere 6 days prior to recurrence constitutes a high‐risk therapeutic error. Notwithstanding an initial positive LA and a high risk of recurrence, the necessity to await a subsequent LA test after 12 weeks should not result in the disregard of a history of thrombosis and a high‐risk profile. A substantial body of research suggests a link between the use of factor Xa inhibitors and an elevated risk of recurrent thrombosis, particularly in patients with triple‐positive or LA‐positive diagnoses [15, 16]. Furthermore, a comprehensive investigation into the etiology of VTE in children is imperative. While obesity is recognized as a risk factor, the occurrence of VTE in children is predominantly associated with more severe underlying diseases, such as systemic lupus erythematosus (SLE) or antiphospholipid syndrome (APS), or with environmental factors. Notably, our case did not involve the use of a central venous catheter (CVC). The patient's hypoalbuminemia and proteinuria are consistent with the findings of Bhasin et al. [13] indicating severe disease activity and a high risk of thrombosis.

The patient exhibited chronic acute PTE, elevated pulmonary artery pressure (PAP > 40 mmHg), and multisystem involvement (kidney, lung, and vascular) in the presence of high‐risk APS, resulting in a CAPS‐like presentation that necessitated long‐term anticoagulation with warfarin and potential lifelong therapy. NOACs are not indicated in this setting.

The effective management of this patient necessitated a series of critical interventions, including the resumption of warfarin and low‐dose aspirin, the administration of steroid pulses to control systemic lupus erythematosus (SLE), care in the pediatric intensive care unit (PICU), and thromboembolectomy for severe pulmonary embolism (PTE). These interventions were instrumental in preserving life and averting organ failure.

## 6 Conclusion

This 14‐year‐old patient with evolving systemic lupus erythematosus (SLE) and triple‐risk antiphospholipid syndrome (APS) developed a severe thrombotic recurrence and chronic acute pulmonary embolism (PTE) after an inappropriate switch from warfarin to rivaroxaban, which was life‐threatening. The patient exhibited a favorable response to the resumption of warfarin in conjunction with low‐dose aspirin, the prompt initiation of pulse methylprednisolone, and management in the pediatric intensive care unit (PICU). Thromboembolectomy was performed to resolve the pulmonary embolism (PTE) and reduce pulmonary artery pressure, resulting in survival and stabilization of the patient. This case underscores the significance of adherence to treatment guidelines, the contraindication of NOACs in patients with APS/LA positivity, and the necessity of specialized interdisciplinary management.

## Author Contributions

**Azadeh Zeinab Mirzaee:** conceptualization, writing – review and editing, writing – original draft. **Bita Paknejad:** investigation. **Aziz Eghbali:** investigation, writing – review and editing. **Reza Shiari:** writing – review and editing. **Niku Rahimi Afzal:** conceptualization, writing – review and editing, writing – original draft.

## Funding

The authors have nothing to report.

## Ethics Statement

This study was conducted in accordance with the principles of the Declaration of Helsinki. Written informed consent was obtained from the patient for both participation in the treatment and the use of their clinical data for research purposes.

## Consent

Written informed consent was obtained from the patient's parents for publication of this case report and any accompanying images. A copy of the written consent is available for review by the Editor‐in‐Chief of this journal.

## Conflicts of Interest

The authors declare no conflicts of interest.

## Data Availability

All data generated or analyzed during this study are included in this published article. Any additional raw data (different original images) are available from the corresponding author upon reasonable request.
